# One minute of stair climbing and descending reduces postprandial insulin and glucose with 3-min improving insulin resistance following a mixed meal in young adults: A Randomized Controlled Crossover Trial

**DOI:** 10.1016/j.jesf.2024.03.004

**Published:** 2024-03-18

**Authors:** Jeff M. Moore, Hannah Salmons, Cameron Vinoskey, Shirin Hooshmand, Jochen Kressler

**Affiliations:** a School of Exercise and Nutritional Sciences, San Diego State University, San Diego, CA, 92182, USA; b The Lundquist Institute for Biomedical Innovation at Harbor-UCLA Medical Center, Torrance, CA, 90502, USA

**Keywords:** Glucose, Insulin, Postprandial, Exercise, Insulin sensitivity

## Abstract

**Background:**

Glycemic markers, including postprandial glucose, insulin, and insulin resistance, are strong predictors of morbidity and mortality in individuals with and without diabetes. Stair-climbing and -descending (SCD) at a comfortable pace for 3 minutes after a sugary beverage (300 kilocalories; 100% carbohydrate) lowers insulin, with insulin sensitivity improving in 10 minutes. If similar benefits are seen following consumption of a mixed meal is unknown. We hypothesize SCD will improve these markers in a dose-response manner following a mixed meal.

**Methods:**

In a randomized, controlled, crossover trial, young adults (N = 31) performed SCD for 0 (seated control), 1, 3, and 10 minutes after a mixed meal (650 kilocalories; 53% carbohydrates, 33% fat, and 14% protein). Differences in glucose, insulin, and insulin sensitivity (ISI) from baseline to 30 min were analyzed using a mixed-effects ANOVA.

**Results:**

A significant fixed-effect was found for change in glucose [F(2.551,67.17) = 4.724,p = 0.007)], insulin [F(2.692,74.49) = 11.28,p < 0.001)], and ISI [F(2.127,56.00) = 5.848,p = 0.004)]. Compared to the seated control (0 minutes), changes in glucose were lower after 1 minute (−14.0 (−7.2)mg/dL,p < 0.001), 3 minutes (−18.4 (−7.0)mg/dL,p = 0.0007), and 10 minutes (−10.0 (−8.1)mg/dL,p = 0.039); changes in insulin were lower after 1 minute (−1.8 (−0.9)μIU/mL,p = 0.0011), 3 minutes (−2.8 (−0.9)μIU/mL,p < 0.001), and 10 minutes (−1.1 (−0.9)μIU/mL,p = 0.033); and changes in ISI were significantly higher after 3 minutes (2.4 (1.5),p < 0.001) and 10 minutes (1.3 (1.6),p = 0.014) but not 1 minute (1.2 (1.5),p = 0.059).

**Conclusion:**

Postprandial glucose and insulin improved with 1 minute, and insulin resistance improved with 3 minutes, of SCD at a self-selected, comfortable pace, after consumption of a mixed meal in apparently healthy young adults.

**Protocol:**

Trial registration: ClinicalTrials.gov Identifier: NCT04232475.

## 1 Introduction

Rates of diabetes mellitus are increasing globally with over 50% of American adults having some form of diabetes.^1^ The majority of these cases are pre-diabetes, of which up to 70% are expected to become type-2 diabetes within their lifetime.^1^^,^^2^ Insulin resistance is the defining characteristic of pre- and type 2 diabetes and is increasingly common in individuals with type 1 diabetes. Positive feedbacks loops make reversal difficult and, without intervention, progression is the norm. The positive feedback loops consist of increased insulin resistance leading to higher insulin levels which in turn increases insulin resistance.^3^

It follows that non-insulin dependent glucose lowering interventions, such as exercise, may be a necessary part of reversing insulin resistance while managing glucose levels. Exercise is an effective means of lowering glucose and improving insulin resistance. Muscular contraction promotes uptake of blood glucose from systemic circulation via signaling molecules including AMPK, NOS, and Ca^2+^ which lead to translocation of non-insulin dependent GLUT-4 transporters.^4^ Individuals with and without diabetes can utilize this process through exercise to acutely lower postprandial blood glucose levels. Adherence to physical activity guidelines is, however, low among the general public with 46%, 72%, and 76% of American adults failing to meet the minimum physical activity guidelines for aerobics, muscle-strengthening, and both, respectively, as of 2018.^5^ Identifying modes of exercise with fewer barriers to participation that are still effective in improving glucose, insulin, and insulin sensitivity is of interest. Previous studies have shown exercise can reduce postprandial blood glucose (PBG), however, they typically used interventions that were relatively long in duration (≥45 minutes) and/or higher in intensity (≥ moderate intensity).^6^^,^^7^ Stair climbing and descending (SCD) is a particularly promising exercise intervention reducing glucose in as little as 1 minute at low intensity.^8^^,^^9^ SCD has additional unique benefits over other modes of exercise that may serve to increase its adoption.

The main barriers to exercise participation include time, perceived effort, intensity, cost, self-efficacy, convenience, equipment, and location.^10^^,^^11^ SCD is a form of exercise for which these barriers are minimal for most people. Previous studies on SCD have found it to be particularly time efficient with bouts as short as 1 minute reducing PBG following a glucose challenge and mixed meal,^8^^,^^9^ bouts as short as 3 minutes reducing insulin after a glucose challenge,^12^ and bouts as short as 10 minutes improving insulin resistance after an oral glucose tolerance test (OGTT) glucose challenge when performed at a self-selected, low to moderate intensity.^12^ SCD reduces postprandial glucose reductions more than cycling or walking when performed for similar durations.^13^^,^^14^ Based on device-measured physical activity monitoring, 10 minutes of SCD was associated with the same magnitude of reduction in glycosylated hemoglobin (HbA1c) as 90 minutes of walking.^15^ SCD is also fairly easy, with perceived exertion being lower than objectively measured intensity.^9^ In a survey of adults living in New York City, 62% of adults reported climbing three or more flights of stairs on an average weekday while 32% reported climbing two or less. Use of stairs was less among women than men, decreased with age, and increased with employment or student status. A study observing nearly 35,000 participants at 8 different locations, including the workplace and an outdoor site, found 72% of males and 58% of females chose to use the stairs when a single elevator was available as an alternative, indicating acceptable self-efficacy among the majority of this sample. When two lifts were available stair use decreased to 54% and 47% in males and females, respectively, suggesting convenience is a significant motivator for use.^16^ Stairs are ubiquitous in many regions of the United States. According to the 2019 American Housing Survey, 47% of US home entrances are inaccessible without climbing up or down stairs.^17^ Residential buildings with stairs as well as other means as access e.g. elevators would not be included in this figure meaning a minimum of 47% of homes have stairs. In addition to in residential buildings, stairs are often found at the workplace and public and commercial buildings. Together, these figures suggest it's reasonable to assume the majority of Americans have access to stairs in their daily life. There is no cost associated with using stairs when they are available.

Whether short SCD bouts at a self-selected pace improve insulin and insulin resistance, as well as PBG, after a mixed meal has yet to be examined. The use of a mixed meal, rather than a glucose challenge, will provide greater ecological validity. We hypothesize that as little as 1 minute of comfortable pace SCD induces significant improvements in glucose, insulin, and insulin resistance and that SCD effects are amplified in a dose-response manner.

## 2 Methods

### 2.1 Subjects

A total of 31 young adults (23.7 (3.0) years; 39% male) were recruited and completed the trial. (Table 1). One participant did not complete the trial due to not tolerating the venipuncture needed to collect venous blood samples. Participants were screened using the Physical Activity Readiness Questionnaire for cardiovascular risks and classified by the American College of Sports Medicine guidelines as low risk for exercise participation.^18^ Written informed consent was obtained from all participants. The trial received approval by the Institutional Review Board at San Diego State University and was conducted in accordance with the Declaration of Helsinki.

**Table 1:** Subject demographics.

	All (N = 31)	Males (n = 12)	Females (n = 19)
Age (years)	23.7 (3.0)	24.9 (3.4)	22.8 (2.7)
Weight (kg)	69.6 (12.6)	80.0 (10.0)	63.7 (8.9)
Height (cm)	170.7 (10.0)	179.3 (8.7)	164.0 (5.9)
BMI (kg/m^2^)	23.8 (3.0)	24.9 (2.8)	23.7 (2.9)
Fasting blood glucose (mg/dL)	91 (11)	93 (13)	90 (9)
Fasting insulin (uU/mL)	14 (8)	13 (9)	15 (8)

Values presented as mean (SD).

### 2.2 Study protocol

Participants performed mixed meal tolerance tests on four separate visits. Visits occurred in the morning between the hours of 8am and 11am after an overnight fast. Each subject was required to schedule their visits at the same time of day (within 1 hour) and between 2 and 7 days apart. Participants were asked to keep the length of their overnight fast constant (within 1 hour) and to consume the same last meal the night before each visit. Instructions were given to maintain their diet and lifestyle habits throughout participation in the study.

Visit order was randomized by investigators using an online random number generator. Each visit contained a mixed meal tolerance test combined with one of the following conditions: seated control (0 minutes of SCD), 1 minute of SCD, 3 minutes of SCD, or 10 minutes of SCD. After baseline measures, participants were asked to consume the test meal within 15 minutes. The time the test meal was finished in was held constant within individuals for every visit. Completion of the meal started the timer for subsequent measures. SCD bouts all ended at minute 28 to allow for venous blood samples to be obtained at minute 30. Participants were asked to choose a "comfortable pace” which was then held constant within individuals for each visit. SCD paces ranged from 90 to 110 steps per minute. SCD were performed in a continuous ascending descending fashion in an indoor 21-step stairwell (step height of 15 cm). As discussed in a previous publication, the subjective intensity of the SCD bouts determined from the modified Borg scale were very light for the 1 and 3 minute and light for the 10 minute conditions.^8^ Participants were monitored during each bout and a metronome was used to ensure adherence to the chosen pace.

All participants received the same test meal and finished it in its entirety within 15 minutes at all visits. The test meal (650 kilocalories) was developed to mimic macronutrient intake of American adults (53% carbohydrates, 33% fat, and 14% protein). The meal included the following: 28g fortified breakfast cereal (Cheerios, General Mills; 240 mL 2% cow's milk (Trader Joe's); one slice (45g) of 100% whole wheat bread (Western Hearth); 20g blackberry jam (Smucker's); 32g peanut butter; and 100 g of ripe banana (Chiquita). Participants were asked to consume the components of the meal in the same order to avoid a meal order effect.^19^^,^^20^ Water intake as allowed ad libitum.

### 2.3 Sample and statistical analyses

Venous samples collected at baseline and minute 30 were centrifuged to collect plasma which was stored at −80 °C until analyses for glucose [Stanbio Glucose Liquicolor] and insulin [Calbiotech Inc., Insulin ELISA Kit] were performed. Glucose and insulin were used in the following formulas to calculate change in glucose, change in insulin, Matsuda's insulin sensitivity index (ISI):

Δ glucose or insulin = 30 minute value - baseline value

ISI:(10,000)/[√(FastingGlucose*FastingInsulin)*(PostprandialGlucose*PostprandialInsulin)]

with glucose expressed as mg/dL and insulin as uU/mL.

Glucose, insulin, and ISI were analyzed using GraphPad Prism 8.0 with a mixed-effects model ANOVA which allowed for inclusion of participants with one or more missing values. The mixed-effects model was used to preserve power and reduce bias from missing values.^21^ An additional assumption of a mixed-effects model is for missing values to be missing at random or completely at random.^21^ This assumption was met as all missing values were due to failed venipuncture or inadequate sample volume for analysis. Post hoc pairwise comparisons were performed with LSD adjustments. Unless stated otherwise, data is presented as mean (SD). Statistical significance was defined as p ≤ 0.05. The current paper is a secondary data analysis of previously published intervention^8^ and thus a power analysis was deemed inappropriate. A separate manuscript assessing sex differences in the acute effect of SCD on PBG^22^ was also published as a secondary analysis of the previously published manuscript.^8^

## 3 Results

### 3.1 Glucose

**Fig. 1:**
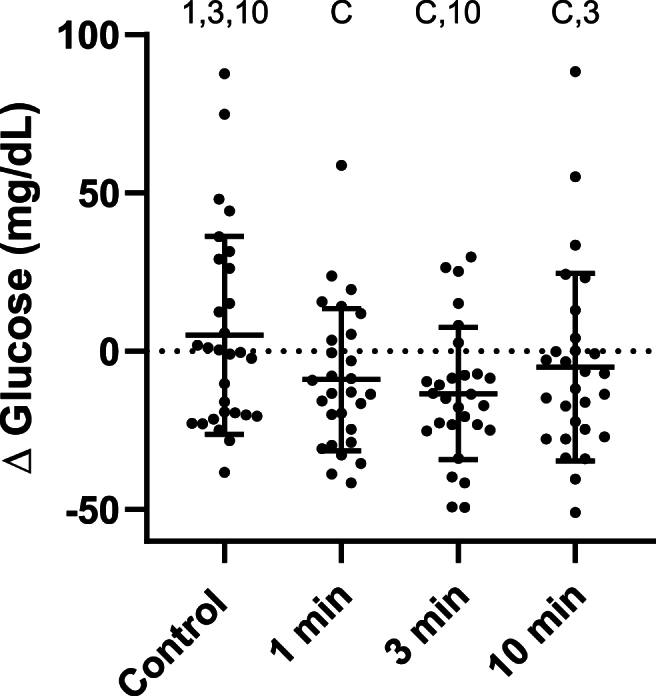
Change in glucose from 0 to 30 min. Each point on the graph is data from a single participant. Bars indicate mean and standard deviation. Symbols indicate a significant difference from that condition (p < .05) C: Seated control; 1: 1 minute SCD; 3: 3 minutes SCD; 10: 10 minutes SCD.

Ten of 124 data points (8%) were missing due to inadequate sample volume or failed venipuncture. Baseline glucose averaged from all conditions was 91 (11) mg/dL with no differences between conditions (F (2.380, 65.86) = 0.4689, p = 0.66). There was a significant fixed effect for change in glucose [F(2.551,67.17) = 4.724, p = 0.007)]. Compared to the control condition, the delta glucose score was lower in the 1 minute SCD (−14.0 (−7.2) mg/dL, p < 0.001), 3 minute SCD (−18.4 (−7.0) mg/dL, p = 0.0007), and 10 minute SCD conditions (−10.0 (−8.1) mg/dL, p = 0.039). The delta glucose score was also lower during the 3 minute SCD compared to the 10 minute SCD conditions (−8.4 (−6.8) mg/dL, p = 0.020). (Fig. 1).

### 3.2 Insulin

**Fig. 2:**
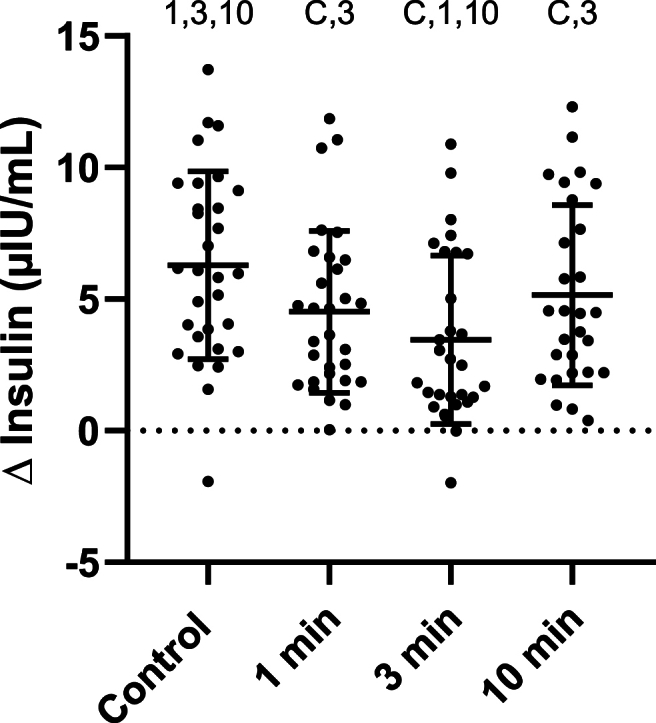
Change in insulin from 0 to 30 minutes. Each point on the graph is data from a single participant. Bars indicate mean and standard deviation. Symbols indicate a significant difference from that condition (p < .05) C: Seated control; 1: 1 minute SCD; 3: 3 minute SCD; 10: 10 minute SCD.

Seven of 124 data points (6%) were missing due to inadequate sample volume or failed venipuncture. Baseline insulin averaged from all conditions was 14 (8) uU/mL with no differences between conditions (F (1.203, 34.07) = 0.2098, p = 0.70). There was a significant fixed effect for change in insulin [F(2.692,74.49) = 11.28, p < 0.001)]. Compared to the control condition the delta insulin score was lower in the 1 minute SCD (−1.8 (−0.9) μIU/mL, p = 0.001), 3 minute SCD (−2.8 (−0.9) μIU/mL, p < 0.001), and 10 minute SCD conditions (−1.1 (−0.9) μIU/mL, p = 0.033). The delta insulin score was also lower during the 3 minute SCD compared to the 1 minute SCD (−1.1 (−0.8) μIU/mL, p = 0.032) and 10 minute SCD (−1.7 (−0.9) μIU/mL, p = 0.001) conditions (Fig. 2).

### 3.3 Insulin sensitivity index (ISI)

**Fig. 3:**
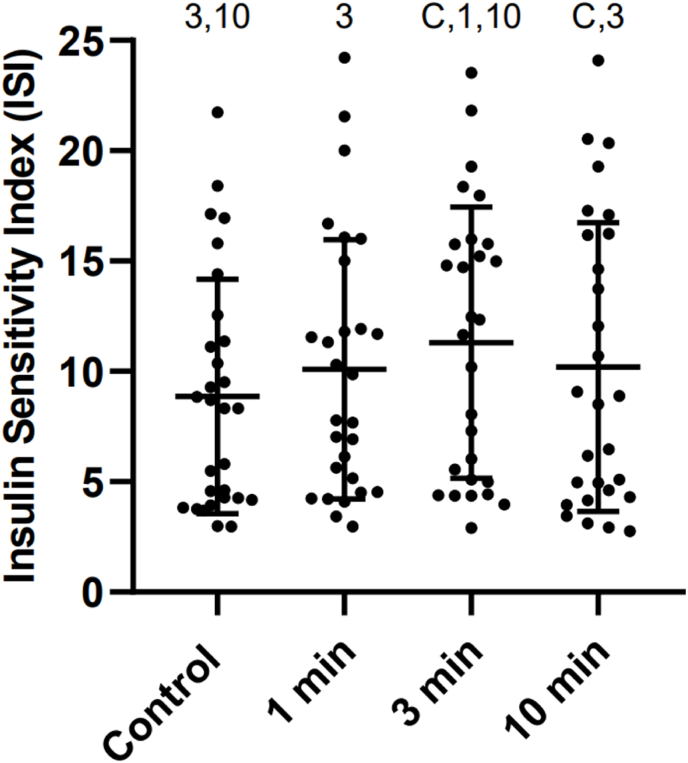
Change in insulin sensitivity index (ISI) from 0 to 30 min. Each point on the graph is data from a single participant. Bars indicate mean and standard deviation. Symbols indicate a significant difference from that condition (p < .05) C: Seated control; 1: 1 min SCD; 3: 3 min SCD; 10: 10 min SCD.

Eleven of 124 data points (9%) were missing due to inadequate sample volume or failed venipuncture. ISI during the control condition was 8.76. There was a significant fixed effect for change in ISI [F(2.127, 56.00) = 5.848, p = 0.004)]. Compared to the control condition, the ISI was significantly higher in the 3 minute SCD (2.4 (1.5), p < 0.001), and 10 minute SCD (1.3 (1.6), p = 0.014) conditions but not the 1 minute SCD conditions (1.2 (1.5), p = 0.059). The ISI was also higher during the 3 minute SCD compared to the 1 minute SCD (1.2 (1.6), p = 0.018) and 10 minute SCD (1.1 (1.7), p = 0.030) conditions (Fig. 3).

## 4 Discussion

We found as little as 1 minute of SCD improves postprandial glucose and insulin after a mixed meal, with at least 3 minutes needed to improve insulin resistance. These results support and expand from previous results that found benefits in glycemic and insulin indices from short SCD bouts after an oral glucose tolerance test.^9^^,^^12^ The replicability of these benefits following short SCD bouts is important as the current paper is the first to assess changes in insulin indices following consumption of a mixed meal which holds more external validity and real world applicability than oral glucose tolerance test drinks containing only simple carbohydrates (dextrose) and no other macronutrients.

Improving insulin resistance by lowering glucose levels via non-insulin dependent pathways, such as exercise, is a crucial component of targeting the alarming increase in diabetes rates, as well as improving risk in non-diabetic individuals. The brevity and perceived intensity (very light to light) of these exercise bouts position SCD as a very appealing option for public health interventions as time and perceived effort are two main barriers to physical activity participation. Other exercise interventions that successfully reduced postprandial insulin used repeated, longer, and/or more intense as well as different modes of exercise. These included single bouts of 24–40 minutes at 50%–83% of peak VO2 and repeated bouts of fifteen 2-minute bouts and two 20-minute bouts at low to moderate intensity.^23^, ^24^, ^25^, ^26^

Comparing the magnitude of insulin attenuation between the current study and others is difficult as other studies are either much higher intensity or longer in overall duration. In the current study, the change from baseline to post exercise relative to the control condition from the 1, 3, and 10 minutes of SCD was −28%, −45%, and −18% respectively. The largest reductions compared to the control condition at any timepoint compared to baseline in a wide range of exercise interventions of similar intensity range vary from −34 to −85%. [26–29]. However, these similar intensity interventions all use much longer duration (30–60 minutes) indicating that SCD may be particularly time effective mode of exercise for insulin reduction.

Studies with shorter duration have much higher intensities. Its previously been shown that 3.3 minutes of very high intensity exercise (all-out sprint on cycle ergometer) can improve ISI in young overweight and obese men (N = 10) compared to a seated control (9.4 (2.1) versus 6.5 (1.3), p = .022), but not insulin (2353 (355) versus 1850 (278), p = .185).^27^ In this trial the oral glucose tolerance tests were conducted the day after the exercise bouts indicating a benefit which persisted at least 18 hours. The improvement in ISI was greater following the 3.3 minute sprint than our 3 minute SCD bout (45% versus 27%). This may be explained by the lower baseline ISI in the overweight and obese participants in their trial allowing for greater room for improvement. This could also suggest a possible ceiling effect and diminished benefit among the healthy participants in our trial and greater possible benefits than seen in our current study among less metabolically healthy individuals.

Very few studies investigated exercise interventions similar to the current study. One study assessing 8 minutes of SCD at a moderate intensity (60–65% heart rate reserve) found no statistically significant change in insulin. Nor was there a change in insulin after the same duration and intensity of cycling. There were reductions in blood glucose after both modes of exercise, with greater reductions following SCD than cycling.^13^ The lack of statistically significant changes in insulin may be a result of low statistical power with only 7 participants. The timing of the exercise, 90 minutes after eating, could also be a factor. Post meal exercise has been shown to be more effective than pre meal exercise for reducing postprandial glucose.^28^ We timed our exercise bouts to occur just before the predicted postprandial glucose peak hypothesizing this strategy would result in a larger reduction in glucose and insulin. Whether the timing of exercise relative to the glucose peak matters should be assessed in future research.

A pilot study found instructing individuals with type 2 diabetes (N = 7) to perform 3 minutes of SCD 60 and 120 minutes after each meal for two weeks improved serum 1,5-anhydroglucitol, a marker of hyperglycemia, but failed to improve HOMA-IR or fasting blood glucose with statistical significance. The lack of statistical significance may be due to lack of necessary statistical power with only 7 participants.^29^ For reasons discussed above it may be more prudent to examine the effects of such interventions on postprandial markers. HOMA-IR in particular does not include postprandial insulin or glucose in its calculation and thus represents hepatic, but not peripheral, insulin resistance.

This study has several limitations. Measures were only obtained at baseline and the anticipated peak of blood glucose (minute 30). Additional sampling would allow for the assessment of the incremental area under the curve for insulin. Measures of diet and lifestyle were not obtained and we instead relied on the randomization design to balance potential differences and accepted the potential increased type 2 error risk from greater variation. All participants received the same size meal which is unlikely to represent every participant's typical meal size. However, because they served as their own control with the crossover design this is unlikely to have impacted our results. Ad libitum water intake could have led to varying intakes which could affect rates of gastric emptying. However, only very large variations of water intake would cause such an issue. The alternative, restricting water intake could have led to hydration status issues if participants ended up not drinking to thirst. Additional variation from differing intakes of water should again be addressed with the randomized crossover design but may have increased our type 2 error risk. In the current study, only the acute effects of SCD were assessed. The effect of repeating these bouts and incorporating them into one's lifestyle should be explored. Participants in the current study were young and in apparent good health. It would be prudent to examine these effects in populations that stand to benefit more, such as individuals with diabetes. Greater effects could be expected in such populations as there would be more room for improvement. Considering other clinical populations could have greater difficulty in performing SCD and some populations should be mindful of fall risk and potential for injury, this intervention is not suitable for all.

## 5 Conclusion

We report the shortest single exercise bouts to improve postprandial glucose, insulin, and insulin sensitivity with only 1 minute, 1 minute, and 3 minutes of stair climbing and descending respectively. The brevity of these exercise bouts in combination with the low self-reported intensity (very light to light) and wide availability of stairs positions stair climbing and descending as a particularly attractive intervention to improve insulin resistance.

## Funding sources

None to report.

## CRediT (contributor roles taxonomy) author statement

Conceptualization: JM, HS, CV, JK. Formal Analysis: JM, JK. Investigation: JM, HS, CV. Methodology: JM, HS, CV, JK, HS. Project administration: JK. Resources: HS. Supervision: JM, SH, JK. Visualization: JM. Writing – original draft: JM. Writing – review & editing: JM, JM, HS, CV, JK. All authors approved the final version of the paper.

## Declaration of competing interest

None to report.
