# Supplementary material for: One minute of stair climbing and descending reduces postprandial insulin and glucose with 3-min improving insulin resistance following a mixed meal in young adults: A Randomized Controlled Crossover Trial
Source: J Exerc Sci Fit. 2024 Mar 18;22(3):266–70. doi: 10.1016/j.jesf.2024.03.004 (PMC10987319; doi:10.1016/j.jesf.2024.03.004)
Supplement: Multimedia component 2 [file mmc2.doc]

**
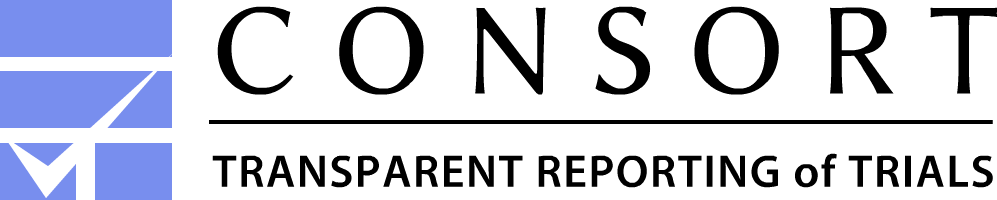
**

**CONSORT 2010 Flow Diagram**

**Allocation**

**Analysis**

**Follow-Up**

**Enrollment**

Assessed for eligibility (n= 32)

Excluded (n= 0)

♦  Not meeting inclusion criteria (n= 0)

♦  Declined to participate (n= 0)

♦  Other reasons (n= 0)

Analysed (n= 31 )
♦ Excluded from analysis (n= 0)

Discontinued intervention (give reasons) (n= 1)

- Did not tolerate venipuncture

dd

- d

Allocated to intervention via crossover RCT (n= 32)

♦ Received allocated intervention (n= 32)

♦ Did not receive allocated intervention (give reasons) (n=0)

Randomized (n= 32)
